# Involvement of salicylic acid, ethylene and jasmonic acid signalling pathways in the susceptibility of tomato to *Fusarium oxysporum*

**DOI:** 10.1111/mpp.12559

**Published:** 2017-05-23

**Authors:** Xiaotang Di, Jo Gomila, Frank L. W. Takken

**Affiliations:** ^1^ Molecular Plant Pathology, Faculty of Science Swammerdam Institute for Life Sciences, University of Amsterdam PO Box 94215, 1090GE Amsterdam the Netherlands

**Keywords:** ET, *Fusarium oxysporum*, JA, SA, susceptibility, tomato

## Abstract

Phytohormones, such as salicylic acid (SA), ethylene (ET) and jasmonic acid (JA), play key roles in plant defence following pathogen attack. The involvement of these hormones in susceptibility following *Fusarium oxysporum* (*Fo*) infection has mostly been studied in *Arabidopsis thaliana*. However, *Fo* causes vascular wilt disease in a broad range of crops, including tomato (*Solanum lycopersicum*). Surprisingly little is known about the involvement of these phytohormones in the susceptibility of tomato towards *Fo* f. sp. *lycopersici* (*Fol*). Here, we investigate their involvement by the analysis of the expression of ET, JA and SA marker genes following *Fol* infection, and by bioassays of tomato mutants affected in either hormone production or perception. *Fol* inoculation triggered the expression of SA and ET marker genes, showing the activation of these pathways. *NahG* tomato, in which SA is degraded, became hypersusceptible to *Fol* infection and showed stronger disease symptoms than wild‐type. In contrast, *ACD* and *Never ripe* (*Nr*) mutants, in which ET biosynthesis and perception, respectively, are impaired, showed decreased disease symptoms and reduced fungal colonization on infection. The susceptibility of the *def1* tomato mutant, and a prosystemin over‐expressing line, in which JA signalling is compromised or constitutively activated, respectively, was unaltered. Our results show that SA is a negative and ET a positive regulator of *Fol* susceptibility. The SA and ET signalling pathways appear to act synergistically, as an intact ET pathway is required for the induction of an SA marker gene, and vice versa.

## Introduction

The root‐infecting fungal pathogen *Fusarium oxysporum* (*Fo*) causes vascular wilt disease in over 100 different plant species, including banana, cotton, palm, Arabidopsis and tomato (Michielse and Rep, 2009). *Fo* represents a species complex comprising many individual pathogenic strains, each capable of infecting one or a few host species only. Based on host specificity, strains have been grouped into *formae speciales*. Infection by *Fo* starts on attachment of fungal hyphae to the plant root surface. Subsequently, fungal hyphae enter the roots through wounds or cracks at the root tip, or at sites of lateral root formation. Ultimately, the fungus reaches the xylem vessels and proliferates, causing disease to ensue (Berrocal‐Lobo and Molina, 2008; di Pietro *et al*., 2003; Rep *et al*., 2002). In attempting to arrest pathogen spread through the vasculature, the plant blocks its infected vessels and compromises their ability to transport water and nutrients. Vascular browning, stunting, progressive wilting and, eventually, plant death are typical disease symptoms of infected plants (Agrios, 2005; di Pietro *et al*., 2003).

In general, plant defence responses against pathogens are controlled by complex signalling routes that often involve the classical defence phytohormones salicylic acid (SA), ethylene (ET) and jasmonic acid (JA) (Robert‐Seilaniantz *et al*., 2011). Usually, SA signalling triggers resistance against biotrophic and hemibiotrophic pathogens, whereas a combination of JA and ET signalling activates resistance against necrotrophs (Glazebrook, 2005).

As a result of the extensive availability of genetic and genomic resources, most studies on phytohormone involvement in defence against *Fo* have been performed in Arabidopsis (Edgar *et al*., 2006). Arabidopsis is susceptible to *Fo forma specialis* (f. sp.) *conglutinans* (*Focn*). Arabidopsis lines that express the salicylate hydroxylase transgene (*NahG*), or that carry the SA *induction‐deficient 2* (*sid2*) mutant, are impaired in SA accumulation. Both lines show increased susceptibility to *Fo*, indicating the involvement of SA in reducing disease susceptibility (Berrocal‐Lobo and Molina, 2004; Diener and Ausubel, 2005).

Pretreatment of Arabidopsis seedlings with either methyl jasmonate (MeJA) or the ET precursor 1‐aminocyclopropane‐1‐carboxylic acid (ACC) leads to enhanced disease symptom development on *Fo* inoculation, indicating that both ET and JA are involved in disease susceptibility (Trusov *et al*., 2009). The ET‐insensitive Arabidopsis mutants *ethylene insensitive2‐1* (*ein2‐1*) and *ethylene receptor 1 (etr1‐1)* show a reduction in disease symptoms compared with Col‐0 plants when inoculated with *Focn* (Pantelides *et al*., 2013; Trusov *et al*., 2009). In contrast, various JA biosynthesis mutants, such as *jasmonate resistant 1* (*jar1‐1*) and *allene oxide synthase* (*aos*), do not exhibit increased susceptibility to *Fo* (Thatcher *et al*., 2009; Trusov *et al*., 2009). Surprisingly, a point mutation in *CORONATINE INSENSITIVE1* (*COI1*), an essential component of JA perception, strongly reduces disease symptom development following *Fo* infection (Thatcher *et al*., 2009; Trusov *et al*., 2009). In addition, disruption of *MYC2*, *PFT1* and *LBD20*, transcriptional regulators of JA signalling, also results in an increased resistance to *Fo* (Anderson *et al*., 2004; Kidd *et al*., 2009; Thatcher *et al*., 2012). Taken together, ET and JA are positive regulators of susceptibility in Arabidopsis.

The role of phytohormones in determining host colonization and disease symptom development is known to vary for different *formae speciales* of *Fo* and their respective hosts (Di *et al*., 2016). To obtain a better insight into these processes, it is therefore crucial to investigate the role of phytohormones in defence responses to *Fo* in plant species other than Arabidopsis. Tomato (*Solanum lycopersicum*), a major and important vegetable crop (Panthee and Chen, 2010), is susceptible to *Fo* f. sp. *lycopersici* (*Fol*), resulting in significant yield losses each year (McGovern, 2015). The interaction between tomato and *Fol* has been well studied (Takken and Rep, 2010). Like other *formae speciales* of *Fo*, *Fol* colonizes the vasculature, and infected plants exhibit vascular browning, leaf epinasty, stunting, progressive wilting and, eventually, death (di Pietro *et al*., 2003). During colonization, the fungus secretes virulence factors, called effector proteins (Houterman *et al*., 2007). The deletion of specific effectors, such as Avr2, typically compromises fungal virulence, resulting in strains that are reduced in pathogenicity (Houterman *et al*., 2007, 2009).

For tomato, a large collection of lines is available which are compromised in hormone perception, metabolism or signalling. In our study, mutants affected in the biosynthesis and signalling pathway of specific defence‐related hormones were analysed for their susceptibility to *Fol*. The lines used in this study include the following: (i) transgenic *NahG* plants that express the bacterial enzyme salicylate hydroxylase, which converts SA into biologically inactive catechol, resulting in plants deficient in SA accumulation (Brading *et al*., 2000); (ii) *Never ripe* (*Nr*), a dominant ET‐insensitive mutant, carrying a single base substitution in the region encoding the N‐terminus of ETR3, a homologue of the Arabidopsis ETR1 receptor (Wilkinson *et al*., 1995); (iii) a transgenic line that expresses *ACCD* (1‐amino‐cyclopropane‐1‐carboxylic acid deaminase), which encodes the ACCd enzyme that catalyses the degradation of ACC; (iv) the JA‐deficient mutant *defenseless‐1* (*def1*), which has a defect in the jasmonate pathway between 13‐hydroperoxy‐octadecatrienoic acid (13‐HPOT) and 12‐oxo‐phytodienoic acid; this mutant fails to produce JA and does not systemically accumulate proteinase inhibitors (PIs) in response to treatment with systemin or oligosaccharide elicitors (chitosan and polygalacturonide) (Li *et al*., 2002); and (v) a *35S::prosystemin* transgenic line overexpressing prosystemin; prosystemin is a positive regulator of JA signalling, and hence these plants constitutively accumulate high levels of PI proteins (Howe and Ryan, 1999).

Here, we report our inoculation assays, using both wild‐type *Fol* and a *FolΔAvr2* mutant, of the various tomato lines affected in SA, ET or JA signalling. In contrast with JA signalling, both SA and ET play major and opposing roles in disease susceptibility and development. The SA and ET signalling pathways appear to act synergistically, as an intact ET pathway is required for the induction of an SA reporter gene, and vice versa. A model for the role of SA, ET and JA signalling in tomato in the susceptibility to *Fol* is proposed and compared with that in Arabidopsis.

## Results

### *NahG* tomato plants show enhanced disease symptom development on *Fol* infection

**Figure 1:**
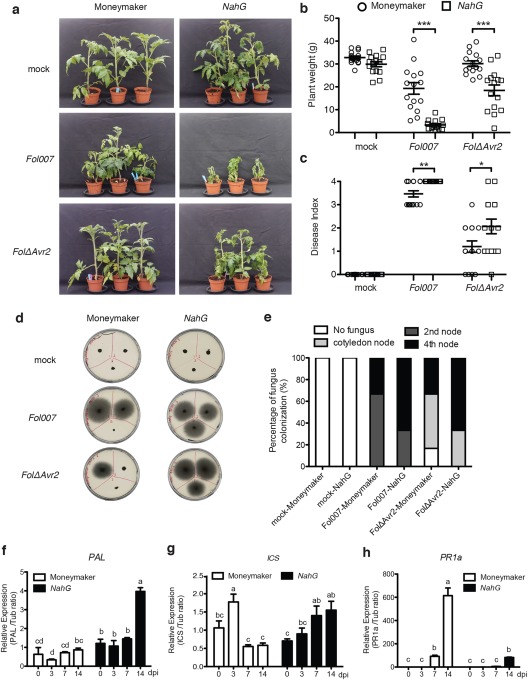
Impaired salicylic acid (SA) signalling enhances *Fusarium oxysporum* f. sp. *lycopersici* (*Fol*) disease symptom development in tomato. (a) Three‐week‐old wild‐type Moneymaker and *NahG* tomato plants inoculated with water (mock), *Fol007* or *FolΔAvr2* at 21 days post‐infection (dpi). Disease development was scored by measuring the fresh plant weight (b) and determining the disease index (range, 0–4) (c) of 20 plants per treatment/genotype combination. Circles and squares indicate Moneymaker and *NahG* plants, respectively. Plant weight was subjected to a pairwise comparison with a Student's *t*‐test, whereas the disease index was analysed by a non‐parametric Mann–Whitney *U*‐test (*P < 0.05; **P < 0.01; ***P < 0.001). The bioassay was repeated three times with similar results. (d) Representative stem sections taken from the cotyledon node (top left), second node (top right) and fourth node (bottom) of individual treated plants (*n* = 6) after incubation for 5 days on potato dextrose agar (PDA) plates. (e) Percentage of infected slices showing fungal outgrowth. Fungal progression in the stem was expressed as the infected percentage of all stem pieces. The experiment was performed twice with similar results. Transcription patterns of *phenylalanine ammonia‐lyase* (*PAL*) (f), *isochorismate* (*ICS*) (g) and *pathogenesis‐related 1a* (*PR1a*) (h) in *Fol007*‐inoculated wild‐type Moneymaker and *NahG* plants at 0, 3, 7 and 14 dpi. Gene expression levels relative to the internal control *tubulin* genes were quantified by quantitative polymerase chain reaction (qPCR). The data are expressed as the mean ± standard deviation (SD). Three biological replicates for each line per time point were analysed. The different letters show significant difference at *P* < 0.05 as determined by Duncan's multiple‐range test. The experiment was performed twice with similar results.

To assess the potential role of SA in the modulation of susceptibility to *Fol*, 3‐week‐old wild‐type tomato plants (cultivar Moneymaker) and transgenic *NahG* plants impaired in SA accumulation were inoculated with either water (mock) or wild‐type *Fol*, notably a race 2 isolate called *Fol007*. In addition, to allow the assessment of hypersusceptibility, a *Fol007 Avr2* knockout strain (*FolΔAvr2*) was included. This mutant is compromised in virulence and causes fewer disease symptoms than wild‐type *Fol* on susceptible plants (Houterman *et al*., 2009). As shown in Fig. 1a, *NahG* plants inoculated with *Fol007* exhibited stronger disease symptoms than did wild‐type plants. These symptoms included extensive wilting and a greater stunting 3 weeks after inoculation. Consistent with this, the fresh weight of *Fol007*‐infected *NahG* tomato plants was significantly lower than that of corresponding wild‐type plants (Fig. 1b). Moreover, all vascular bundles of infected *NahG* plants had turned brown, and plants were either dead or very small and wilted. On a scale from 0–4 (Rep *et al*., 2005), infected *NahG* plants scored the maximal disease index (Fig. 1c). As expected, *FolΔAvr2‐*inoculated plants developed weaker disease symptoms (Fig. 1a). Similar to *Fol007*, *FolΔAvr2*‐infected *NahG* plants showed a significant reduction in fresh weight and a higher disease index relative to infected wild‐type plants (Fig. 1b,c).

To investigate whether the augmented disease symptom development in *NahG* plants correlated with increased host colonization, a fungal recovery assay was performed. Sections were taken from *Fol*‐inoculated wild‐type and *NahG* plants at different heights of the stem, notably at the position of the cotyledon node, second node and fourth node. Following sterilization, the sections were placed on potato dextrose agar (PDA) plates and incubated for 5 days at 25 °C. As shown in Fig. 1d,e, wild‐type *Fol* colonized the stems more extensively than did the *FolΔAvr2* strain. In all cases, the wild‐type fungus was able to reach the second or even fourth node, whereas, in the *Avr2* knockout, only 30% reached the second node and the majority of the fungus was contained at the cotyledon node or below. Notably, the *FolΔAvr2* strain was able to more effectively colonize *NahG* plants, and fungal outgrowth was observed in the fourth node in 70% of the inoculations (Fig. 1e). The *NahG* plants were also hypersusceptible to the wild‐type fungus, as depicted by the higher percentage of plants in which fungal outgrowth was seen from the fourth node. Together, these data suggest that *NahG* plants are hypersusceptible to *Fusarium* infection, and that the increased disease symptoms correlate with increased fungal colonization of the transgenic plants.

SA is synthesized through both the isochorismate (ICS) and phenylalanine ammonia‐lyase (PAL) pathways (Lee *et al*., 1995; Wildermuth *et al*., 2001). To assess the expression of the *PAL* and *ICS* genes during *Fol* infection, a reverse transcription‐quantitative polymerase chain reaction (RT‐qPCR) analysis was carried out on hypocotyls. Samples were taken at 0, 3, 7 and 14 days post‐infection (dpi) of wild‐type and *NahG* plants. *PAL* and *ICS* expression levels were measured and normalized to tubulin. We found that, in *NahG* plants, only at 14 dpi transcript levels of *PAL* were significantly up‐regulated relative to that at 0 dpi (Fig. 1f). In the wild‐type Moneymaker plants, no significant induction of *PAL* was observed at any time point. *ICS* expression in *NahG* plants was significantly induced at 7 and 14 dpi relative to that at 0 dpi (Fig. 1g). In the wild‐type plants, a significant, but only transient, induction of *ICS* was observed at 3 dpi. Together, these data indicate the involvement of SA biosynthesis genes in the *Fol*–tomato interaction. Possibly, the high SA turnover in *NahG* plants elicits a feedback mechanism activating the PAL and ICS SA biosynthesis pathways following *Fol* infection.

*Pathogenesis‐related 1a* (*PR1a*) expression is often used as a reporter for SA‐dependent defence signalling (Kunkel and Brooks, 2002). To assess whether *PR1a* expression is altered during *Fol* infection, its expression at 0, 3, 7 and 14 dpi was measured. Transcript levels of *PR1a* were significantly induced in wild‐type plants at 7 and 14 dpi, suggesting that SA signalling is activated late during *Fol* infection (Fig. 1h). Compared with wild‐type plants, the expression of *PR1a* in infected *NahG* plants was less strongly induced. This weaker induction correlates with the loss of SA accumulation in *NahG* plants, confirming the proposed of the transgenic line. Overall, these data show that impaired SA signalling enhances susceptibility to *Fol* and disease symptom development in tomato.

### ET enhances susceptibility to *Fol* in tomato plants

Pretreatment of Arabidopsis seedlings with the ET precursor ACC leads to enhanced disease symptom development on *Focn* inoculation, indicating that ET is involved in disease susceptibility (Trusov *et al*., 2009).

**Figure 2:**
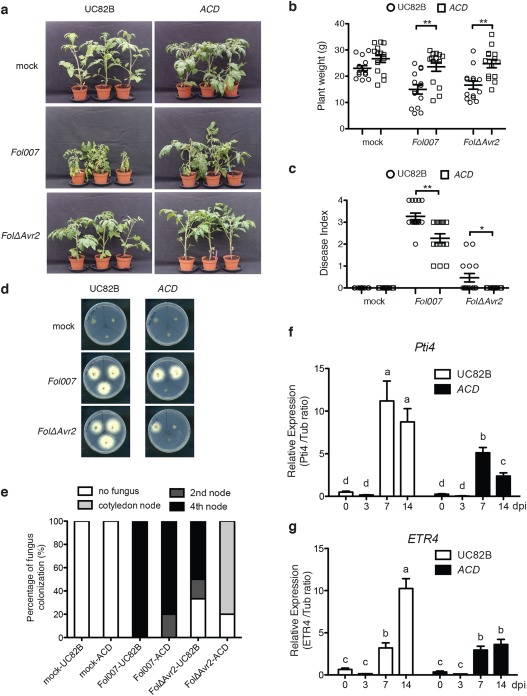
Impaired ethylene (ET) biosynthesis and production in tomato reduces disease susceptibility to *Fusarium oxysporum* f. sp. *lycopersici* (*Fol*). (a) Three‐week‐old wild‐type UC82B and *ACD* tomato plants inoculated with water (mock), *Fol007* or *FolΔAvr2* at 21 days post‐infection (dpi). Disease development was scored by measuring the fresh weight (b) and disease index (range, 0–4) (c) of 20 plants per treatment/genotype combination. Circles and squares indicate UC82B and *ACD* plants, respectively. Plant weight was subjected to a pairwise comparison using a Student's *t*‐test, whereas the disease index was analysed by a non‐parametric Mann–Whitney *U*‐test (*P < 0.05; **P < 0.01; ***P < 0.001). The bioassay was repeated three times with similar results. (d) Representative stem sections taken from the cotyledon node (top left), second node (top right) and fourth node (bottom) of individual treated plants (*n* = 6) after incubation for 5 days on potato dextrose agar (PDA) plates. (e) Colonization is expressed as the percentage of infected slices of all stem pieces (*n* = 6). The experiment was repeated twice with similar results. (f, g) Transcription patterns of ET‐regulated marker genes *Pti4* and *ETR4* in *Fol007*‐inoculated UC82B and *ACD* at 0, 3, 7 and 14 dpi. Gene expression levels relative to the internal control *tubulin* genes were quantified by quantitative polymerase chain reaction (qPCR). The data are expressed as the mean ± standard deviation (SD). Three biological replicates for each line per time point were analysed. The different letters show the significant difference at *P* < 0.05 as determined by Duncan's multiple‐range test. The experiment was performed twice with similar results.

To investigate the role of ET in disease symptom development in tomato, transgenic plants impaired in ET biosynthesis were analysed for their susceptibility to *Fol*. In the transgenic *ACD* line, constitutively expressing a bacterial ACC deaminase gene, ET production is reduced by 90% relative to the wild‐type (Klee *et al*., 1991). As the transgene is present in cultivar UC82B, this cultivar was used as wild‐type control. Although wild‐type UC82B showed severe wilting and stunting following *Fol* infection, most *ACD* plants showed only mild disease symptoms (Fig. 2a). The weight of infected *ACD* plants was also significantly higher than that of infected wild‐type plants (Fig. 2b). In addition, the disease index in *ACD* plants was significantly attenuated relative to that in wild‐type plants (Fig. 2c). A similar reduction in symptom development was also observed in *ACD* lines inoculated with *FolΔAvr2*. To monitor fungal colonization, stem sections were taken and incubated on PDA plates. The fungal recovery assay showed that *Fol007* grew‐out from most stem sections of both wild‐type and *ACD* plants, whereas much less fungal growth was observed in *FolΔAvr2‐*inoculated *ACD* plants. A typical example of a plate assay is shown in Fig. 2d and the data from the fungal recovery assay are summarized in Fig. 2e. *FolΔAvr2* was found to efficiently colonize wild‐type UC82B plants, as fungal outgrowth was often observed up to the fourth node. In contrast, colonization of *ACD* plants was much reduced: in 80% of cases, the fungus was only observed in stem sections collected at the cotyledon node. These data indicate that the *ACD* line exerts a reduced susceptibility towards *Fol* infection concomitant with a reduction in symptom development.

It has been reported that the ET receptor gene *ETR* and ET‐responsive factors *ERF*s are induced following *Fo* infection (Berrocal‐Lobo and Molina, 2008; Pantelides *et al*., 2013). A well‐characterized ERF family member in tomato is Pti4, which has been shown to specifically bind to the GCC‐box *cis*‐element present in the promoter of *PR* genes (Wu *et al*., 2002). GCC‐box binding by ERFs induces *PR* gene expression and *Pti4* could be a functional tomato homologue of *ERF1* (Wu *et al*., 2002). In Arabidopsis, *ERF1* overexpression enhances resistance to *Fo* (Berrocal‐Lobo and Molina, 2004). As shown in Fig. 2f,g, the transcription of *Pti4* and *ETR4* was strongly induced in wild‐type plants at 7 and 14 days, respectively, following *Fol* inoculation. Compared with wild‐type plants, *Fol*‐infected *ACD* plants showed a weaker increase in expression of the two ET marker genes, which confirms the proposed plant genotypes. In addition, the weaker induction of *ETR4* and *Pti4* expression in the *ACD* line is a further indication that ET‐mediated signalling is activated during *Fol* infection.

**Figure 3:**
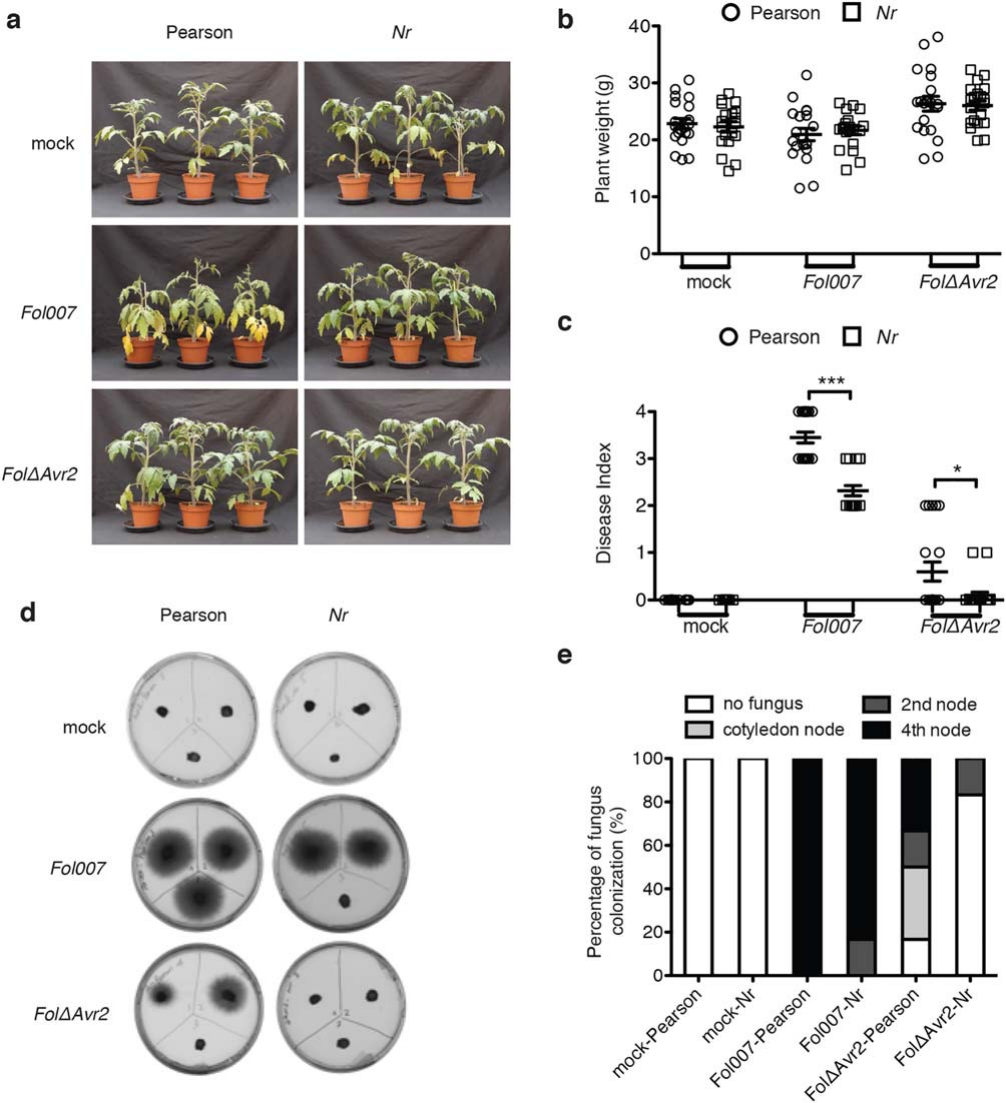
Ethylene (ET) perception is required for *Fusarium oxysporum* f. sp. *lycopersici* (*Fol*) disease symptom development in tomato. (a) Three‐week‐old wild‐type and *Never ripe* (*Nr*) Pearson tomato plants inoculated with water (mock), *Fol007* or *FolΔAvr2* at 21 days post‐infection (dpi). Disease symptoms were scored by measuring the fresh weight (b) and disease index (range, 0–4) (c) of 20 plants per treatment/genotype combination. Circle and square indicate Pearson plant and *Nr* plant, respectively. Plant weight was subjected to a pairwise comparison using a Student's *t*‐test, whereas the disease index was analysed by a non‐parametric Mann–Whitney *U*‐test (*P < 0.05; **P < 0.01; ***P < 0.001). The bioassay was repeated three times with similar results. (d) Representative stem sections taken from the cotyledon node (top left), second node (top right) and fourth node (bottom) of individual treated plants (*n* = 6) after incubation for 5 days on potato dextrose agar (PDA) plates. (e) Colonization is expressed as the percentage of infected slices of all stem pieces (*n* = 6). The experiment was repeated twice with similar results.

To further test how ET signalling contributes to increased susceptibility, the involvement of ET perception by the host was investigated. Bioassays were performed with wild‐type tomato cultivar Pearson and the ET‐insensitive Pearson mutant *Nr*. Following the inoculation of wild‐type Pearson with *Fol007*, the older leaves of infected plants became chlorotic and the plants showed mild wilting symptoms (Fig. 3a). On inoculation with *FolΔAvr2*, wild‐type Pearson plants showed hardly any symptoms (Fig. 3a). Notably, no obvious disease symptoms were observed in *Nr* plants inoculated with either *Fol007* or *FolΔAvr2*. Although the fresh weight of *Nr* plants was identical to that of Pearson plants after *Fol007* infection (Fig. 3b), the *Nr* plants exhibited a significantly lower disease index than Pearson plants; fewer brown vessels were observed in the stems (Fig. 3c). Fungal recovery assays revealed that *FolΔAvr2* either completely failed to colonize *Nr* plants or, in the rare cases it did, it only reached the basal part of the stem that forms the hypocotyl (Fig. 3d,e). Taken together, the data suggest that both the ability to synthesize ET and the ability to perceive the hormone are essential for disease development and the ability of the fungus to colonize the plant.

### Perturbation of JA signalling has no detectable effect on plant susceptibility and disease symptom development

In Arabidopsis, various JA biosynthesis mutants, such as *jasmonate resistant 1* (*jar1‐1*) and *allene oxide synthase* (*aos*), do not exhibit increased susceptibility to *Focn* (Thatcher *et al*., 2009; Trusov *et al*., 2009). To investigate the role of JA biosynthesis in the susceptibility of tomato to *Fol*, we used the *def1* mutant. This line has a defect in its octadecanoid biosynthesis pathway, which provides precursors for JA synthesis, making the plant hypersusceptible to herbivores because of its impaired accumulation of PIs I and II in response to wounding (Lightner *et al*., 1993). In addition to *def1*, *35S::prosystemin* plants were included in our assays. In these plants, the *prosystemin* gene is constitutively overexpressed by the 35S cauliflower mosaic virus promoter. Prosystemin is the precursor of systemin, which initiates a signalling pathway that leads to the synthesis of JA from linolenic acid (Ryan, 2000). The constitutive induction of the JA pathway in *35S::prosystemin* plants results in the systemic accumulation of high levels of PIs in these plants (McGurl *et al*., 1994).

To confirm the genotype of *def1* and *35S::prosystemin* plants, the transcript levels of *PI‐I* were examined in leaves of wild‐type Castlemart, *def1* and *35S::prosystemin* plants in response to wounding. As shown in Fig. S1 (see Supporting Information), wounding of wild‐type plants triggered the induction of *PI‐I* expression. In contrast, no *PI‐I* accumulation was observed on wounding of *def1* plants, whereas a constitutive expression of *PI‐I* was observed in *35S::prosystemin* plants irrespective of treatment. These results confirm the plant genotypes and the involvement of *def1* and *35S::prosystemin* in JA signalling.

**Figure 4:**
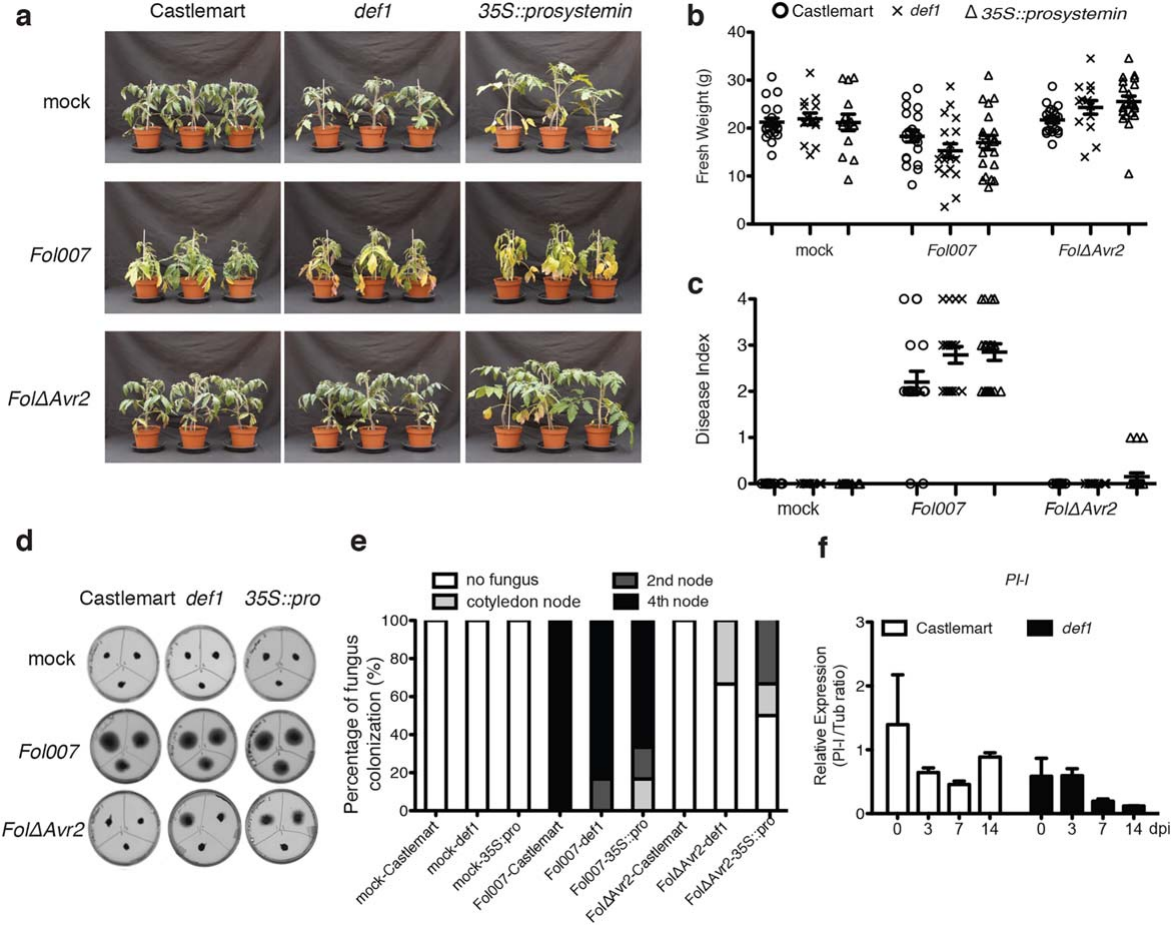
Perturbation of jasmonic acid (JA) signalling has no detectable effect on plant susceptibility. (a) Three‐week‐old wild‐type Castlemart, *def1* and *35S::prosystemin* tomato plants inoculated with water (mock), *Fol007* or *FolΔAvr2* at 21 days post‐infection (dpi). Disease symptoms were scored by measuring the fresh weight (b) and disease index (range, 0–4) (c) of 20 plants per treatment/genotype combination. Circle, × and Δ indicate Castlemart, *def1* and *35S::prosystemin*, respectively. The bioassay was repeated three times with similar results. (d) Representative stem sections taken from the cotyledon node (top left), second node (top right) and fourth node (bottom) of individual treated plants (*n* = 6) after incubation for 5 days on potato dextrose agar (PDA) plates. (e) Colonization is expressed as the percentage of infected slices of all stem pieces (*n* = 6). The experiment was repeated twice with similar results. (f) Transcription patterns of *proteinase inhibitor I* (*PI‐I*) in *Fol007*‐inoculated wild‐type Castlemart and *def1* at 0, 3, 7 and 14 dpi. Three biological replicates for each line were analysed. The data are expressed as the mean ± standard deviation (SD). The experiment was performed twice with similar results.

To test whether JA signalling affects the susceptibility to *Fol*, the JA mutant lines were inoculated with the fungus. As shown in Fig. 4a, *Fol007*‐infected *def1* and *35S::prosystemin* lines became equally chlorotic as the wild‐type Castlemart. Inoculation with the less pathogenic *FolΔAvr2* strain did not result in obvious disease symptoms in any of the lines. No significant differences in fresh weight or disease index were observed between Castlemart and its derivatives following either *Fol007* or *FolΔAvr2* inoculation (Fig. 4b,c).

Fungal recovery assay revealed that, in wild‐type Castlemart, *Fol007* was present in all probed stem sections, showing that the fungus colonized the stem until the fourth node (Fig. 4d,e). In most of the inoculated *def1* and *35S::prosystemin* plants, *Fol007* had also colonized the entire stem until the fourth node. In contrast with *Fol007*, *FolΔAvr2* completely failed to colonize wild‐type Castlemart plants, which is consistent with the lack of disease symptoms observed in these plants. However, in the *35S::prosystemin* plants and *def1* mutants, the *FolΔAvr2* strain was detected at a limited number of stem sections collected at either the position of the cotyledon node or the second node (Fig. 4e). The latter data suggest that disturbed JA signalling might facilitate fungal colonization.

To assess whether JA signalling is induced following *Fol007* inoculation, the expression of *PI‐I* was monitored at 0, 3, 7 and 14 dpi. As shown in Fig. 4f, no significant difference in *PI‐I* expression was observed in *Fol*‐inoculated wild‐type plants at any time point. The same low levels of *PI‐I* expression were detected in the *def1* mutant, and these levels did not change during infection. Taken together, these data imply that perturbation of JA signalling has no detectable effect on the susceptibility to *Fol*.

### SA and ET signalling pathways act synergistically in tomato susceptibility to *Fol* infection

**Figure 5:**
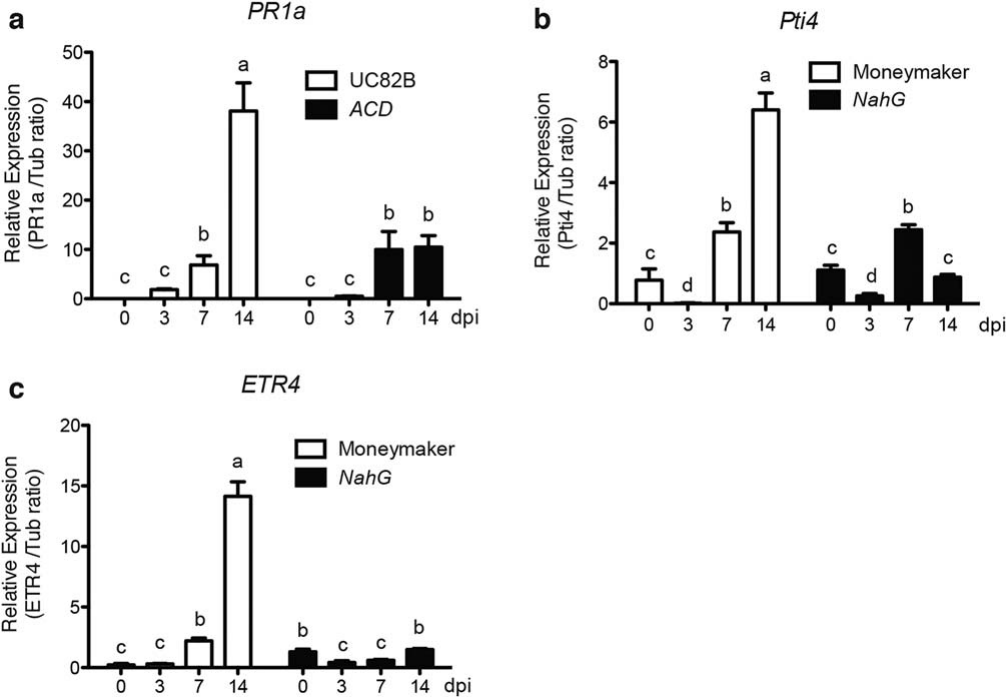
Time course of transcription patterns of salicylic acid (SA) and ethylene (ET) marker genes on *Fusarium oxysporum* f. sp. *lycopersici* (*Fol*) inoculation. (a) Expression of the SA marker gene *pathogenesis‐related 1a* (*PR1a*) in wild‐type UC82B and the transgenic *ACD* line at 0, 3, 7 and 14 days post‐infection (dpi). (b, c) Transcription patterns of the ET marker genes *Pti4* and *ETR4* in *NahG* and the Moneymaker progenitor at 0, 3, 7 and 14 dpi. Gene expression levels relative to the internal control *tubulin* genes were quantified by quantitative polymerase chain reaction (qPCR). The data are expressed as the mean ± standard deviation (SD). Three biological replicates for each line per time point were analysed. The different letters show the significant difference at *P* < 0.05 as determined by Duncan's multiple‐range test. The experiment was performed twice with similar results.

The degradation of SA was found to enhance the susceptibility to *Fol*, whereas impaired ET signalling reduced the susceptibility. We therefore wanted to test whether these pathways interact following *Fol* infection. The expression of *PR1a* was monitored over time (0–14 dpi) in both the *ACD* ET synthesis mutant and in wild‐type UC82B plants on *Fol007* infection. As shown in Fig. 5a, expression of the SA marker gene *PR1a* was strongly induced in wild‐type plants, but much less so in *ACD* lines. This finding indicates that the induction of *PR1a* following *Fol* infection requires an intact ET pathway. Subsequently, the expression of *Pti4* and *ETR4* was assessed in the *NahG* line and its parental Moneymaker background following *Fol007* inoculation. As shown in Fig. 5b,c, expression of both ET marker genes was strongly induced in wild‐type plants, but not in *NahG* plants, following *Fol* infection. This finding suggests that an intact SA signalling pathway is required for *Pti4* and *ETR4* induction.

## Discussion

Here, the role of SA, ET and JA in the modulation of the susceptibility of tomato plants to *Fol* was investigated. *NahG* plants that failed to accumulate SA were hypersusceptible to *Fol* infection and showed severe disease symptoms and extensive fungal colonization of their xylem vessels. Together with the strong induction of the SA marker gene *PR1a* in wild‐type plants, which was not strongly induced in *NahG* plants, these data show that SA plays a positive role in reducing disease susceptibility. This conclusion is in agreement with chemical studies, in which exogenous application of SA to tomato through root feeding or foliar sprays reduced vascular browning, leaf yellowing and wilting following *Fol* inoculation (Mandal *et al*., 2009). The positive role of SA in *Fol* resistance in tomato is consistent with studies with *NahG* Arabidopsis, showing an increased susceptibility to *Fo* (Berrocal‐Lobo and Molina, 2004; Diener and Ausubel, 2005; Thatcher *et al*., 2009; Trusov *et al*., 2009). Therefore, the role of SA in *Fo* susceptibility seems to be conserved in both plant species. Interestingly, in several studies, elevated SA has been reported to enhance the susceptibility to necrotrophic pathogens, but to promote resistance to hemibiotrophs (Bari and Jones, 2009; El Oirdi *et al*., 2011). Our findings, in which SA reduces the susceptibility of tomato plants to *Fol*, are in line with the hemibiotrophic lifestyle of the latter (Di *et al*., 2016).

The *ACD* tomato line, in which ET biosynthesis is compromised, showed a reduced susceptibility to *Fol* infection. On *Fol* infection, the transgenic line showed fewer disease symptoms and a reduced fungal colonization relative to the wild‐type UC82B cultivar (Fig. 2). The ET marker genes *ETR4* and *Pti4* were highly induced in wild‐type plants, but not in the *ACD* line, indicating that ET signalling is induced in response to *Fol* infection and is important for disease development. The ET‐insensitive *Nr* mutant was also found to be less susceptible than wild‐type Pearson plants to infection with *Fol007* (Fig. 3). These data are consistent with studies by Lund *et al*. (1998) and Francia *et al.* (2007), which also reported a reduction in *Fol* disease symptoms in the *Nr* mutant, The fact that both ET synthesis and perception were found to be important for disease development and fungal colonization suggests that ET signalling is important for full susceptibility to *Fol*.

The role of ET is multifaceted in Arabidopsis (Di *et al*., 2016). Similar to *Nr* in tomato, soil‐grown *ein2‐1* and *etr1‐1* mutants of Arabidopsis showed a reduction in disease symptoms relative to wild‐type Col‐0 plants when inoculated with *Focn* (Pantelides *et al*., 2013; Trusov *et al*., 2009). *Fo* inoculation of Arabidopsis plants carrying the *ein2‐5* allele revealed a markedly enhanced susceptibility to *Fo* in plate assays (Berrocal‐Lobo and Molina, 2004). These differences might be explained by the different mutations, or by the different inoculation methods. As inoculation of soil‐grown plants best mimics the natural infection process, which resembles that of our tomato assays, it seems that, in both plant species, the role of ET is conserved, in that its absence reduces disease symptom development (Pantelides *et al*., 2013; Trusov *et al*., 2009).

No significant difference in disease index and fresh weight between the wild‐type and JA‐deficient *def1* line was found after *Fol007* or *FolΔAvr2* inoculation (Fig. 4). These findings contrast those of Thaler *et al*. (2004), who showed that the weight of *def1*, but not wild‐type tomato plants, was reduced on inoculation with a race 1 *Fol* isolate. The reason for the discrepancy might be that a different *Fusarium* race or different assay conditions were used. On *Fol007* infection, *35S::prosystemin* plants also did not show a significant difference in disease index or fresh weight relative to wild‐type tomato. Thus, under our assay conditions, JA does not appear to play a major role in the development of disease symptoms or disease susceptibility.

In different host–*Fo* pathosystems, JA can promote either resistance or susceptibility (Di *et al*., 2016). A point mutation in *COI1* in Arabidopsis, an essential component for JA perception, strongly reduces the susceptibility to *Fo* (Thatcher *et al*., 2009; Trusov *et al*., 2009). In contrast, *jar1* mutants, which are defective in the synthesis of the bioactive JA–isoleucine (JA‐Ile) conjugate, show wild‐type‐like symptoms or only a slight increase in susceptibility (Thatcher *et al*., 2009; Trusov *et al*., 2009). In addition, Cole *et al*. (2014) have reported that infection by *Focn* and *Fo* f. sp. *matthioli*, which produce JA‐Ile and leucine‐conjugated JA (JA‐Leu), respectively, is suppressed in *coi1*. In contrast, the *coi1* mutation has no effect on infection by *Fo* f. sp. *raphani*, which produces no detectable JA‐Ile/JA‐Leu. Furthermore, the JA‐insensitive mutation (*jai1*) has no effect on the infection of tomato plants by *Fol*, which produces no detectable jasmonates. Therefore, different *formae speciales* may adopt different strategies to infect their host and to cause disease symptoms.

The SA, ET and JA signalling pathways are entangled in a complex network in which the different pathways influence each other through positive and negative regulatory interactions (Grant and Jones, 2009). We observed that, relative to wild‐type plants, the expression of *PR1a* was less strongly induced in the ET biosynthesis mutant *ACD* (Fig. 5). Similarly, following *Fol* inoculation, ET signalling, as monitored by *ETR4* and *Pti4* expression, was strongly induced in wild‐type plants, but less so in *NahG* plants. Collectively, these results indicate that, in tomato, SA and ET signalling might act synergistically during *Fol* infection, as an intact ET pathway is required for the induction of the SA reporter gene, and vice versa. In addition, for *Xanthomonas campestris* pv. *vesicatoria* infection of tomato, accumulation of SA was found to require ET synthesis, suggesting that ET positively regulates SA‐induced defences (O'Donnell *et al*., 2003).

**Figure 6:**
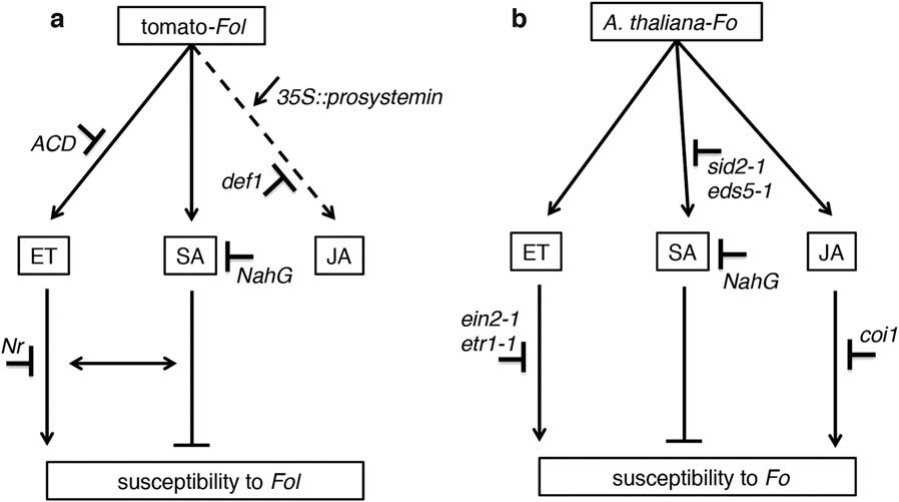
Proposed models for the involvement of jasmonic acid (JA), salicylic acid (SA) and ethylene (ET) signalling in tomato (a) and Arabidopsis (b) on *Fusarium oxysporum* (*Fo*) infection. Compromised ET biosynthesis and perception reduce disease susceptibility, whereas compromised SA signalling promotes hypersusceptibility to *Fo* f. sp. *lycopersici* (*Fol*) infection in tomato. The SA and ET pathways act synergistically, in that induction of one pathway requires the intactness of the other. Although, in Arabidopsis, the JA pathway is required for disease susceptibility, it is not in tomato. By convention, the arrowhead implies positive regulation (stimulation) and the T‐bar implies negative regulation.

Altogether, these data allow us to propose a model for the involvement of SA, ET and JA signalling in tomato in susceptibility to *Fol* (Fig. 6a). SA and ET signalling interact and have opposite roles in disease susceptibility. The infection of tomato plants by *Fol* activates both the ET and SA pathways. The ET response enhances the susceptibility to *Fol* infection and disease development, whereas SA responses restrict colonization. No apparent role for JA was shown in the interaction, as disease symptoms in JA mutants were indistinguishable from those of the wild‐type plants. A comparison with the reported roles of these phytohormones in Arabidopsis in *Fo* infection reveals shared and unique effects between Arabidopsis and tomato. As shown in Fig. 6b, SA signalling also negatively regulates susceptibility to *Fo*, whereas ET signalling likewise positively enhances susceptibility. Notably, JA can be hijacked by the fungus to enhance pathogenicity in Arabidopsis, but apparently does not play an important role in tomato.

Although the Arabidopsis–*Fo* system serves as a useful model for some plant–*Fo* interactions, the tomato system can be used to study *Fo*–plant interactions in which JA does not play a major role. It will be interesting to also investigate other plant–*Fo* systems to identify whether there may be more species‐specific aspects that differ from either model. These insights are relevant to allow the translation of molecular mechanisms obtained in these models into various crops to aim for a reduced susceptibility to wilt disease. The molecular mechanisms underlying susceptibility, however, are currently unknown and their elucidation is a challenge for future studies.

## Experimental Procedures

Ten different tomato (*Solanum lycopersicum*) genotypes were used in these studies, including the four wild‐type cultivars from which these mutants were derived: Moneymaker, UC82B, Pearson and Castlemart. The transgenic *NahG* line, which is compromised in SA accumulation, is in a Moneymaker background (Brading *et al*., 2000). The ET‐impaired mutant *ACD* (Klee *et al*., 1991) and the *Nr* mutation (Lanahan *et al*., 1994) are in a UC82B and Pearson background, respectively. The JA‐impaired mutant *def1* (Howe *et al*., 1996) and the prosystemin‐overexpressing line (*35S::prosystemin*) are in the Castlemart background (McGurl *et al*., 1994). Tomato seedlings were grown in a conditioned glasshouse with day–night temperatures of 23 °C–18 °C and a 16‐h light/8‐h dark regime.

### *Fusarium* inoculation assay

Wild‐type *Fusarium* strain *Fol007* (race 2) and the derived *FolΔAvr2* mutant have been described previously (Houterman *et al*., 2009). *Fol* strains were grown on PDA (Oxoid Ltd., Basingstoke, Hampshire, UK) at 25 °C for 7–10 days. Subsequently, a piece of agar carrying the fungus was transferred to 100 mL minimal medium (100 mm KNO_3_, 3% sucrose and 0.17% yeast nitrogen base without amino acids or ammonia). Conidial spores were harvested after 3–5 days of cultivation at 25 °C with shaking. After washing with sterilized water, the spores were diluted to 10^6^ spores/mL. For bioassay, 3‐week‐old tomato seedlings were uprooted from the soil. The seedlings were placed for 5 min in the *Fol* spore suspension (10^6^ spores/mL) and subsequently potted. Disease progression was evaluated after 3 weeks. Plant weight and disease index (Rep *et al*., 2005) were scored for 20 plants/treatment.

### Fungal recovery assay

Fungal colonization in tomato plants was assessed at 21 dpi. Representative stem sections taken from the cotyledon node, second node and fourth node were collected separately. The stem pieces were surface sterilized in 70% ethanol and rinsed in sterile distilled water, after which the ends of the stems were removed with a sterile scalpel. Stem sections about 5 mm thick were cut and placed on PDA supplemented with 200 mg/L streptomycin and 100 mg/L penicillin at 25 °C, allowing the fungus to grow out of the stem sections. Photographs were taken after 5 days of incubation at 25 °C. Data were expressed as a percentage of slices showing fungal outgrowth.

### Wounding assay

Wounding experiments have been descripted previously (Howe *et al*., 1996). Briefly, a haemostat was used to inflict damage to the primary leaf of 15‐day‐old tomato plants. Twenty‐four hours later, the wounded leaf and an upper unwounded leaf were dissected and snap frozen in liquid N_2_ to use for expression analysis.

### Analysis of gene expression by RT‐qPCR

RNA isolation and cDNA synthesis were performed as described previously (Gawehns *et al*., 2014). Briefly, total RNA from tomato stem beneath the cotyledon was extracted using Trizol‐Reagent (Invitrogen, Life Technologies, Grand Island, NY, USA) according to the manufacturer's instructions. The RNA was subsequently purified with an RNeasy Mini kit (Qiagen, Düsseldorf, Germany) and DNA was removed by on‐column treatment with RNase‐free DNase (Qiagen). cDNA was synthesized using the M‐MulV reverse‐transcriptase RNase H minus kit (Fermentas, Thermo Scientific, Pittsburgh, PA, USA). Stem tissue was collected from tomato plants at 0, 3, 7 and 14 dpi. The conditions of the RT‐qPCR experiments and the relative quantification of specific mRNA levels were performed according to Lopez‐Raez *et al*. (2010) and using the gene‐specific primers described in Table S1 (see Supporting Information). PCRs were performed in an ABI 7500 Real‐Time PCR system (Applied Biosystems, http://www.appliedbiosystems.com Foster City CA, USA) using the Platinum SYBR Green qPCR SuperMix‐UDG kit (Invitrogen). The 20‐μL PCRs contained 0.25 μm of each primer, 0.1 μL ROX reference dye and 1 μL of cDNA. The cycling programme was set to 5 min at 50 °C, 5 min at 95 °C, 40 cycles of 15 s at 95 °C and 1 min at 60 °C, followed by a melting curve analysis. The expression levels of selected genes were normalized to tomato α‐tubulin (Solyc04g077020.2) expression. Relative gene expression was calculated using the 2^–Δ^ ^*CT*^ method. Three biological replicates for each of the selected genes were performed.

### Statistical analyses

The statistical significance of the results was determined by performing PRISM 5.0 (GraphPad, http://www.graphpad.com). The data on plant weight were subjected to a pairwise comparison with Student's *t*‐test. For data on the disease index in tomato plants, a Mann–Whitney test was performed for each genotype. Gene expression data were statistically evaluated, and bars annotated with different letters were significantly different according to Duncan's multiple‐range test.

## Supporting information

Additional Supporting Information may be found in the online version of this article at the publisher's website.

**Fig. S1** The expression of *proteinase inhibitor I* (*PI‐I*) was examined by reverse transcription‐polymerase chain reaction (RT‐PCR) in unwounded (u) and wounded (w) leaves of Castlemart, *def1* and *35S::prosystemin*. Two biological replicates for each line were analysed.

**Table S1** Primer sequences used in the gene expression analysis.
