# Supplementary figures and images for: Involvement of salicylic acid, ethylene and jasmonic acid signalling pathways in the susceptibility of tomato to Fusarium oxysporum
Source: Mol Plant Pathol. 2017 May 23;18(7):1024–35. doi: 10.1111/mpp.12559 (PMC6638294; doi:10.1111/mpp.12559)

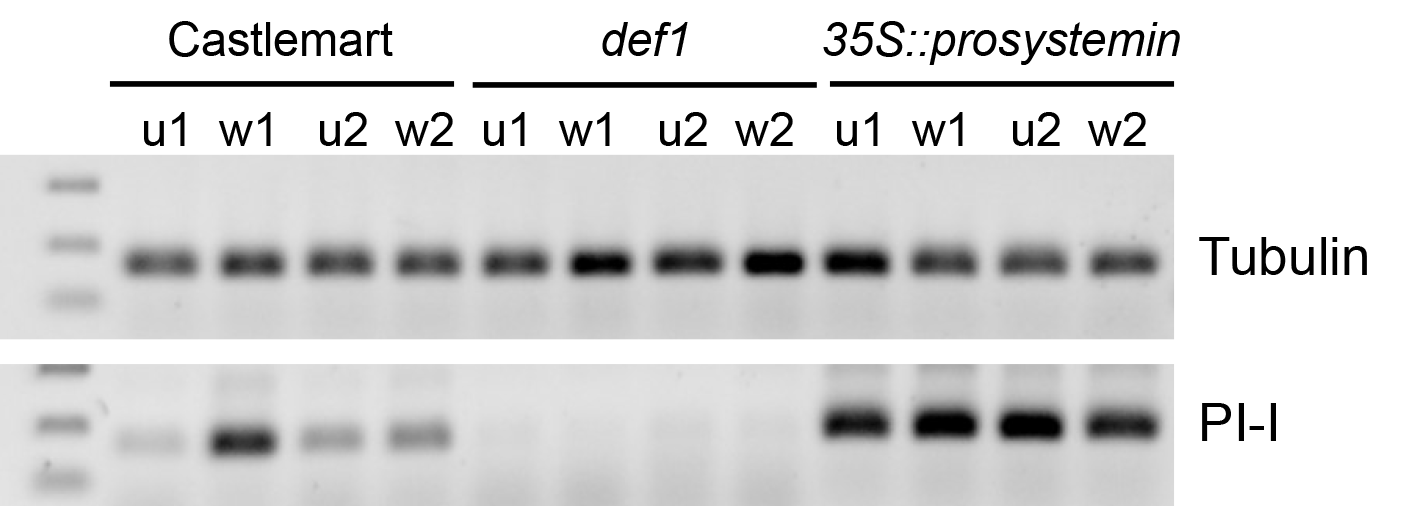

Supplement: Supplementary file 1 — Fig. S1 The expression of proteinase inhibitor I (PI‐I) was examined by reverse transcription‐polymerase chain reaction (RT‐PCR) in unwounded (u) and wounded (w) leaves of Castlemart, def1 and 35S::prosystemin. Two biological replicates for each line were analysed. [file MPP-18-1024-s001.tif]
