# Supplementary material for: Involvement of salicylic acid, ethylene and jasmonic acid signalling pathways in the susceptibility of tomato to Fusarium oxysporum
Source: Mol Plant Pathol. 2017 May 23;18(7):1024–35. doi: 10.1111/mpp.12559 (PMC6638294; doi:10.1111/mpp.12559)
Supplement: Supplementary file 2 — Table S1 Primer sequences used in the gene expression analysis. [file MPP-18-1024-s002.docx]

Supplemental Table1. Primer sequences used in the gene expression analysis.

| Gene | Forward primer (5’-3’) | Reverse primer (5’-3’) |
| --- | --- | --- |
| α-tubulin | TCGTGGCCACTATACCATTG | AGTGACCCAAGACCTGAACC |
| PR1a | TGGTGGTTCATTTCTTGCAACTAC | ATCAATCCGATCCACTTATCATTTTA |
| Pti4 | TCGTCGGGAAACGGTTCCAT | GACATCCAACTTGCATGACACTTG |
| ETR4 | GGTAATCCCAAATCCAGAAGGTTT | CAATTGATGGCCGCAGTTG |
| PAL | CGTTATGCTCTCCGAACATC | GAAGTTGCCACCATGTAAGG |
| PI-I | TCCAGGCTGAAGATGATGAG | TTATTCCAACCGCAAATTCA |
| ICS | TCCAGGCTGAAGATGATGAG | TTATTCCAACCGCAAATTCA |
